# Stress-Relieving Effects of Green Tea Depend on the Ratio of Its Special Ingredients and the Infusion Conditions

**DOI:** 10.3390/molecules29194553

**Published:** 2024-09-25

**Authors:** Keiko Unno, Kyoko Taguchi, Tomoko Matsuda, Yoriyuki Nakamura

**Affiliations:** 1 Tea Science Center, University of Shizuoka, 52-1 Yada, Shizuoka 422-8526, Japan; gp1719@u-shizuoka-ken.ac.jp (K.T.); yori.naka222@u-shizuoka-ken.ac.jp (Y.N.); 2 Mie Prefecture Agricultural Research Institute Green Tea Industry Flower and Planting Laboratory, 992-2 Tsubakise-cho, Kameyama City 519-0104, Japan; matsut39@pref.mie.lg.jp

**Keywords:** stress, theanine, caffeine, epigallocatechin gallate, arginine, green tea, infused solution

## Abstract

Theanine, an amino acid unique to tea leaves, has been reported to exhibit stress-relieving effects. However, the stress-relieving effects of theanine (T) are greatly inhibited by caffeine (C) and epigallocatechin gallate (E), the main components of green tea, while being enhanced by arginine (A). Animal and clinical studies using matcha tea have shown that it can alleviate stress if the molar ratio of C and E against T and A (CE/TA) is less than 2. In general, the CE/TA ratio in tea leaves is reflected by the CE/TA ratio in the infused solution; however, it is not clear what infusion conditions would be expected to ensure a stress-relieving effect. In this study, to examine the stress-relieving effect of green-tea-infused solutions, the CE/TA ratio was measured under different infusion conditions. Furthermore, a study using a model solution consisting of C, E, T, and A revealed that a CE/TA ratio of at least less than 2 and a T content greater than 0.23 mM were required for stress reduction. Based on these results, we identified tea leaves and their infusion conditions that can be expected to reduce stress.

## 1. Introduction

Theanine, an amino acid unique to tea leaves, is synthesized by theanine synthase from glutamic acid and ethylamine in the roots of the tea plant and migrates through the stem to new shoots as the plant grows [1]. Once transferred to the leaves, theanine is rapidly metabolized to glutamate and ethylamine, and the ethylamine-derived carbon is intensively converted to catechins. However, under shading, this metabolism to catechins is severely inhibited. Thus, while shading prevents theanine decline, it also inhibits catechin synthesis.

Green tea is usually made by collecting immature shoots. This is because as maturity progresses, free amino acids such as theanine decrease, and the umami taste decreases. However, green tea that is shaded with black cheesecloth for 2–3 weeks before harvest maintains a high content of free amino acids, resulting in green tea with a high umami taste. Green tea made from new shoots grown in sunlight is called “Sencha” and is high in catechins and low in amino acids. Coarse tea, or “Bancha”, is generally made from hard shoots and old leaves and contains much less amino acids.

Theanine has been reported to exhibit relaxant [2] and stress-relieving effects [3]. However, the stress-relieving effects of theanine (T) are greatly inhibited by the presence of caffeine (C) and epigallocatechin gallate (EGCG, E), which are major components of green tea, while arginine (A), the second most abundant amino acid in tea after T and glutamine, has been found to cooperatively improve T’s effects [4]. Therefore, we propose that the stress-relieving effect of green tea can be predicted by calculating the ratio of the molar sum of T and A, which have stress-relieving effects, to the molar sum of C and E, which have counteracting effects [5]. In animal and clinical studies using matcha green tea, we have shown that a ratio of C and E to T and A (CE/TA ratio) of 2 or less is sufficient to alleviate stress [5]. In addition, we have shown that commercially available matcha teas have varying CE/TA ratios [5].

Among the amino acids other than T and A in green tea, aspartic acid and glutamic acid—which are known to act as excitatory neurotransmitters [6]—have no suppressive effects on stress response, but the stress-reducing effect of T was cancelled with the addition of asparagine and aspartic acid [7]. However, the effect of aspartic acid is not as strong as the antagonistic effect of C and E on T [7]. It has also been confirmed that epigallocatechin (EGC), the second most abundant catechin after EGCG, does not counteract T [4]. Based on these findings, it is reasonable to use the CE/TA ratio in tea leaves and infused solutions as an index for evaluating the stress-reducing effect of green tea, in terms of the suppression of stress response.

The ratio of these components in tea leaves varies greatly depending on the growing conditions, cultivar, harvest time, and part of the leaf to be harvested [8]. In addition, even when the same green tea is used, the ratio of components in the eluate varies depending on the infusion conditions [9]. For example, E and C elute well in hot water but decrease in cold water. On the other hand, amino acids and catechins without a gallate group, such as EGC and epicatechin (EC), elute well even in cold water. Although the infusion rate of each component in green tea eluate solution varies depending on the conditions, the CE/TA ratio in tea leaves is generally reflected in that of the infused solution. However, the infusion conditions under which the stress-relieving effects of tea leaves can be expected have not yet been clarified. In this study, we aimed to clarify the relationship between the CE/TA ratios in tea leaves and the associated infused solutions. For this purpose, the amounts of each component and CE/TA ratios were determined when tea leaves with different CE/TA ratios were infused at various water temperatures and times. Furthermore, as green teas with CE/TA ratios of 2 or higher are generally more common, we investigated whether these teas could reduce stress through modifying the infusion conditions.

An initial response to stress is the activation of the HPA (hypothalamus–pituitary–adrenal) axis, causing the body to initially enter a state of heightened excitation when experiencing stress [10,11,12,13,14]. As continued overexcitement during stress can lead to undesirable changes in various biological responses [15,16,17,18,19], it is important to suppress stress-induced overexcitement as an initial countermeasure to stress. In this study, we evaluated the suppressive effects of green tea on the relatively early stress response in male mice using confrontation rearing as an experimental model.

## 2. Results

### 2.1. Tea Components in Tea Leaves

Five types of green tea were used, denoted from sample A to sample E, which were predicted to differ mainly in T content. The amount of T increases when tea leaves are shaded before harvest. Therefore, three types of shaded tea were selected, differing in the tea cultivars and the way they were shaded. The tea samples A and B (cultivars “Seimei” and “Yabukita”, respectively) were grown under 85% shading for 10 days and 98% shading for 5 days starting from the two-leaf stage. Sample C (“Yabukita”) was shaded with 85% light for 15 days from the three-leaf stage. The other two teas (samples D and E) were regular “Yabukita” Sencha green tea, grown under normal sunlight, with different leaf hardnesses. Sample D was made from soft new leaves, while Sample E was coarse tea with hard new leaves and old leaves used as raw materials.

The amount of T differed depending on the tea cultivar and the method of shading and was higher in the shaded tea leaves. After T, the amino acids with the highest contents were glutamine, A, aspartic acid, glutamic acid, serine, and asparagine, in that order. This order was generally the same in all tea samples. C levels were also higher in the shaded tea leaves than in the unshaded ones. Catechins, on the other hand, were higher in the unshaded tea leaves than in the shaded leaves. In “Seimei” tea leaves, E was lower than in “Yabukita” tea leaves. The molar ratios of C and E to T and A in each green tea are shown in Table 1.

### 2.2. Stress-Relieving Effects of Tea Leaf Powder Consumption

The evaluation of stress-relieving effects can be determined according to whether a test substance can suppress hypertrophy of the adrenal glands through the activation of the HPA axis under stress-loading conditions, which can be assessed in terms of the territoriality of male mice [20]. When two male mice are kept alone in a partitioned cage for about one week, they establish a sense of territoriality. Then, the partitions are removed, and the two mice are housed in the same cage, both mice are subjected to stress from the presence of other mice in their territory (i.e., confrontation stress). Aggressive and defensive behaviors are sometimes observed in these mice.

**Figure 1:**
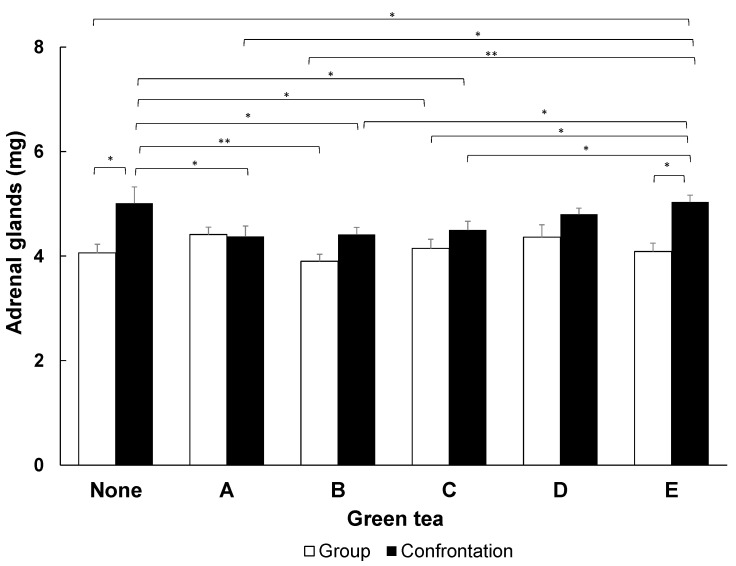
Inhibition of adrenal hypertrophy in mice fed tea leaf powder. The wet weight of the adrenal glands of mice that had been confrontation-reared for 1 day was compared between groups of group-reared mice and groups of mice that had ingested tea leaves with different CE/TA ratios. Bars in each column represent means ± SE (n = 8) (* *p* < 0.05 and ** *p* < 0.01, Fisher’s least significant difference method).

In the control group, the adrenal glands were significantly enlarged in mice under the stressful conditions of confrontation rearing, compared to those in the group-reared mice (Figure 1). On the other hand, mice fed green tea with CE/TA ratios of 1.33, 1.98, and 2.74 (samples A, B, and C) had significantly suppressed adrenal gland hypertrophy, even under the stressful conditions of confrontation rearing. On the other hand, the green tea samples with CE/TA ratios of 5.80 and 14.58 (samples D and E) did not suppress adrenal hypertrophy.

Green tea samples A–C contained more than 18 mg T/g green tea. This is almost consistent with the amount of T required to reduce stress in an animal experiment using matcha green tea, which was 17 mg/g or greater [21]. The intake of powdered green tea by mice was about 100 mg/kg, indicating that the required amount of T for stress reduction was more than 1.8 mg/kg.

### 2.3. Tea Components in the Infused Solution

**Figure 2:**
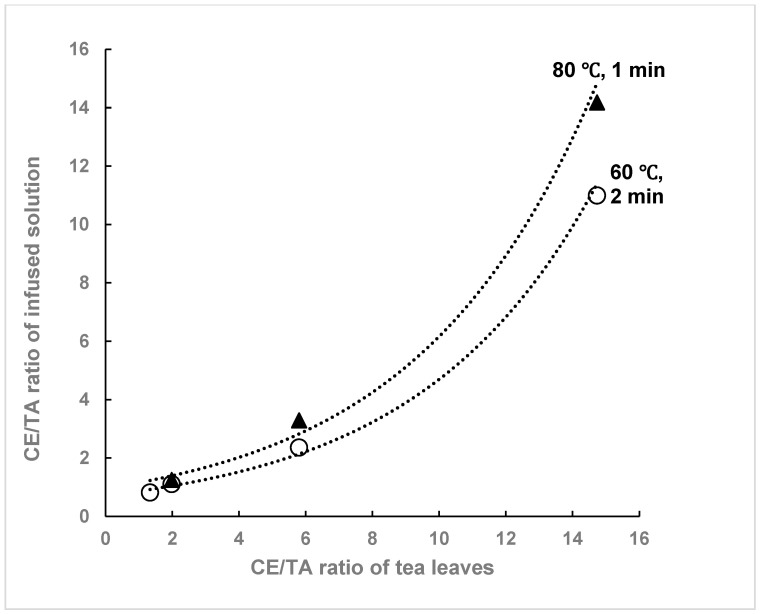
Relationship between CE/TA ratio in tea leaves and CE/TA ratio in infused solutions. Five grams of each tea leaf was infused in 200 mL of water at 60 °C for 2 min (○) or 80 °C for 1 min (▲).

The concentrations of amino acids, catechins, and C in tea-infused solutions and CE/TA ratios were compared for green teas with different CE/TA ratios. The concentrations of each component in the infused solutions obtained under the same infusion conditions (5 g tea leaves infused in 200 mL of hot water at 60 °C for 2 min) are shown in Table 2. Green tea with a CE/TA ratio of 1.98 (sample B) had CE/TA ratios of 1.11 and 1.25 when infused at 60 °C and 80 °C, respectively, and both infused solutions were expected to have a stress-reducing effect (Table 2, Figure 2). On the other hand, green tea with a CE/TA ratio of 5.8 (sample D) had a CE/TA ratio of 2.36 when infused at 60 °C and, thus, can be expected to be effective in reducing stress; however, when infused at 80 °C, the CE/TA ratio was 3.29 and, in this case, the stress reduction effect was not expected.

T, A, and C in the infused solutions were higher for the shaded teas (samples A and B) than the non-shaded regular tea leaves (samples D and E), while the content of E did not significantly differ between these green tea eluates. From the values in Table 1 and Table 2, the infusion efficiency of each component at 60 °C can be determined. The CE/TA ratio in the infused solution reflected that of the tea leaves under the same infusion conditions (Figure 2), but there were slight differences in the elution efficiency of each tea component (Table 3). The elution efficiency of T and C was significantly lower in sample E (CE/TA 14.58), while that of E did not differ much (Table 3). The elution efficiency of sample E was lower than those of the other samples, probably as sample E was a coarse tea made from hard new leaves and old leaves. The CE/TA ratio in the infused solution was lower than that of the tea leaves; however, for tea leaves with higher CE/TA ratios, the CE/TA ratio in the infused solution approached that of the tea leaves as the infusion temperature increased (Figure 2).

**Figure 3:**
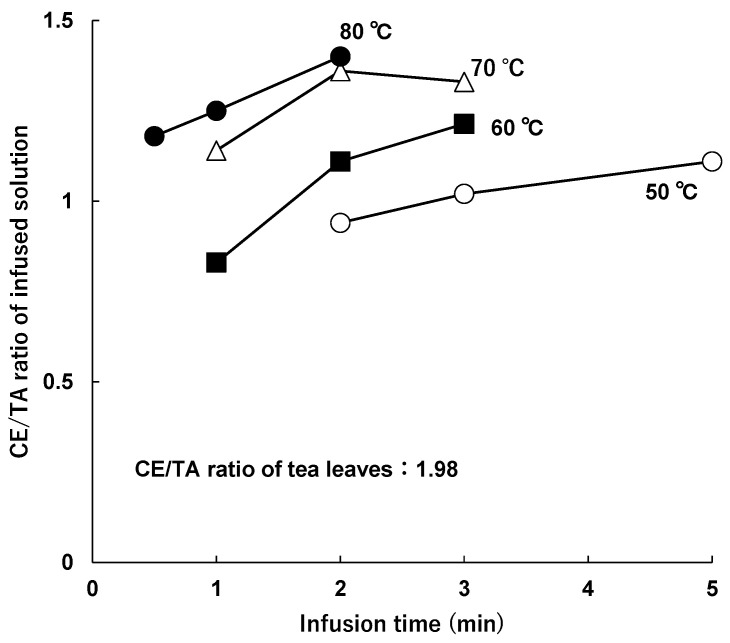
Change in CE/TA ratio with infusion time. Tea leaves (2.5 g) with a CE/TA ratio of 1.98 (sample B) were infused with 100 mL of water at 50 °C (○), 60 °C (■), 70 °C (△), or 80 °C (●) for each infusion time.

The CE/TA ratio in the infused solutions increased with increasing infusion time at the same water temperature (Figure 3). At 60 °C, the CE/TA ratio was 0.83 after 1 min of elution, whereas it was increased to 1.21 after 3 min of elution.

**Figure 4:**
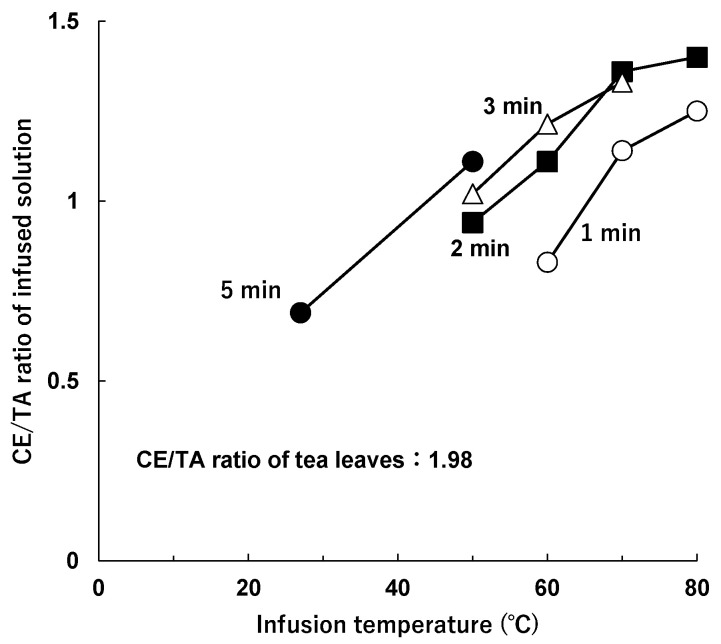
Change in CE/TA ratio as a function of infusion temperature. Tea leaves (2.5 g) with a CE/TA ratio of 1.98 (sample B) were infused in 100 mL of water at various temperatures for 1 min (○), 2 min (■), 3 min (△), or 5 min (●).

Furthermore, at the same infusion time, the higher the water temperature wase, the higher the CE/TA ratio was in the infused solution (Figure 4). Even with an infusion time of 2 min, the CE/TA ratio in the infused solution was 0.94 at 50 °C but increased to 1.4 at 80 °C.

On the other hand, when 2.5 g of tea leaves were infused in 100 mL of water at 60 °C for 2 min, the CE/TA ratio in the eluate was 1.11 (Table 4). When the amount of tea leaves was increased to 5 g, the CE/TA ratio was 1.09, and the amount of each component, including T, was almost doubled (Table 4). Thus, changing the amount of tea leaves relative to the amount of water had little effect on the CE/TA ratio.

**Figure 5:**
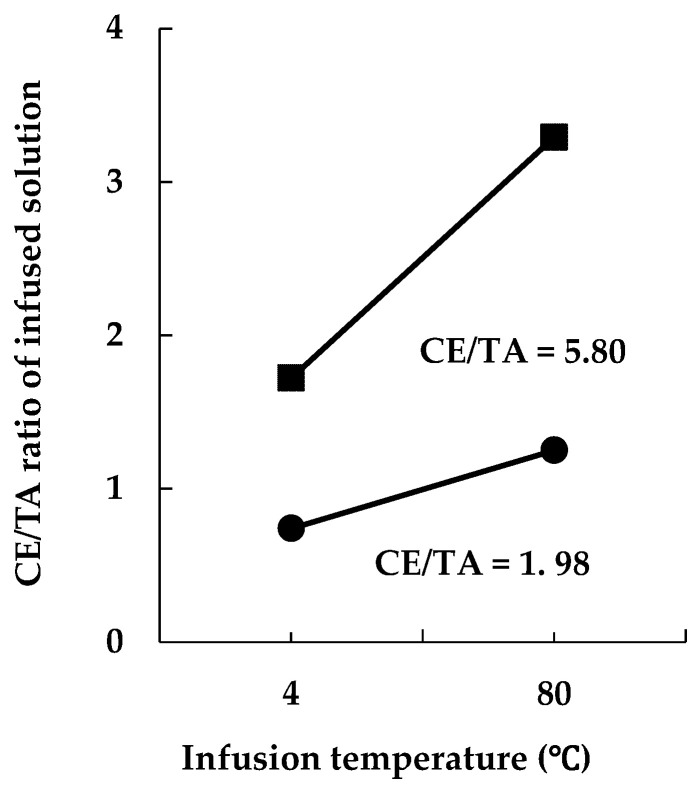
CE/TA ratio was lowered when samples were infused in cold water. Five grams of tea leaves with CE/TA ratios of 1.98 (●; sample B) and 5.80 (■; sample D) were infused in 200 mL of water at 4 °C for 15 h, or 2.5 g of tea leaves in 200 mL of water at 80 °C for 1 min.

The CE/TA ratio decreased when the tea leaves were infused at 4 °C, and the CE/TA ratio in the infused solution was less than 2 even for tea leaves with a CE/TA ratio of 5.8 (sample D) (Figure 5). As twice as many tea leaves were used in the 4 °C elution as in the 80 °C elution, the amino acids in the eluate and the non-gallate catechins (i.e., EGC and EC) increased by approximately 1.5-fold (Table 5). On the other hand, the elution of E and C was suppressed; E and ECG were about 30%, and C was about 44% of the predicted values when 5 g of tea leaves were eluted at 80 °C (Table 5). These results indicate that the water temperature used for infusion has a significant effect on the CE/TA ratio in the eluate.

### 2.4. Evaluation of Stress-Relieving Effects of Model Green Tea Ingredients

Next, we confirmed that the stress reduction effect of the green-tea-infused solutions could be properly evaluated according to the CE/TA ratio based on the four components T, A, C, and E. Their concentrations in the model green tea were set as shown in Table 6, referring to the values of these components in the infused solution detailed in Table 2.

**Figure 6:**
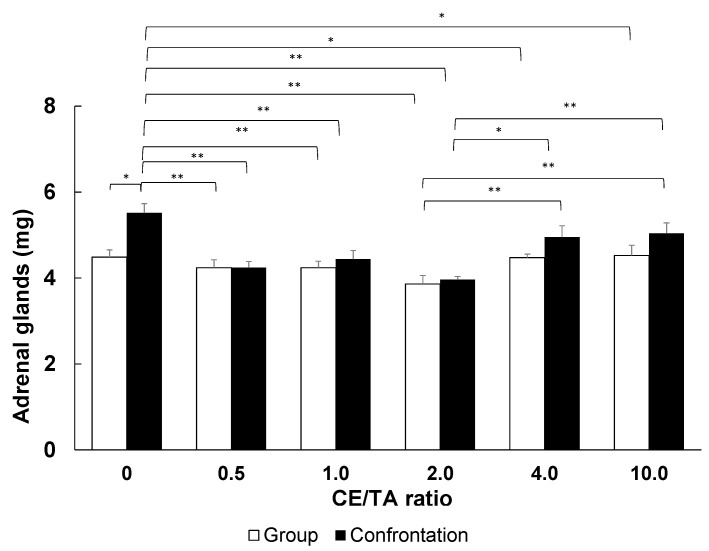
Inhibition of adrenal hypertrophy in mice fed model green tea. The wet weight of the adrenal glands of mice that had been confrontation-reared for 1 day was compared between groups of group-reared mice and groups of mice that had ingested the model green tea with different CE/TA ratios. Bars in each column represent means ± SE (n = 8) (* *p* < 0.05 and ** *p* < 0.01, Fisher’s least significant difference method).

Stress exposure through confrontation rearing significantly suppressed adrenal hypertrophy in the model green tea intake group with CE/TA ratios ranging from 0.5 to 2.0, with a minimum at a CE/TA ratio of 2.0 (Figure 6). In the group-rearing, adrenal hypertrophy tended to be suppressed in the model green tea intake group with CE/TA ratios of 0.5 to 2.0. As the mice were not completely stress-free, even under group-rearing conditions, it is likely that drinking the model green tea solution reduced their stress. On the other hand, adrenal hypertrophy was not suppressed in the model green tea intake group with a CE/TA ratio of 4.0 or higher.

**Figure 7:**
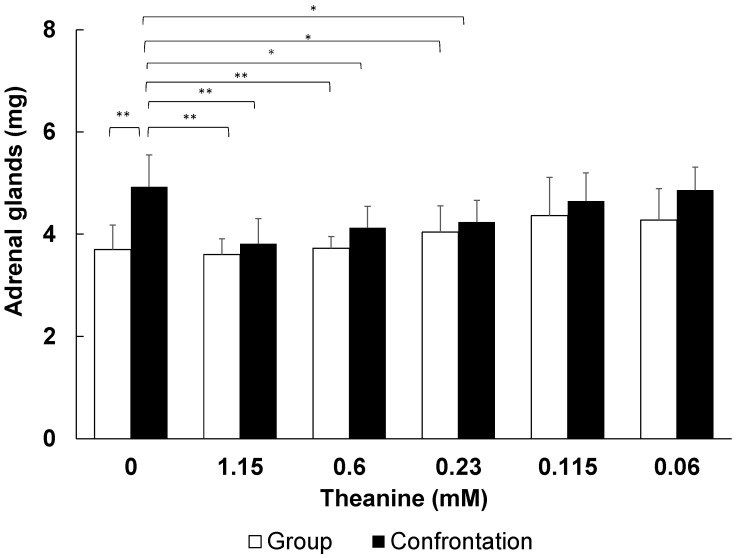
Inhibition of adrenal hypertrophy in mice fed model green tea with CE/TA ratio of 2.0. The wet weight of the adrenal glands of mice that had been confrontation-reared for 1 day was compared between groups of group-reared mice and groups of mice that had ingested the model green tea with different T contents. Bars in each column represent means ± SE (n = 8) (* *p* < 0.05 and ** *p* < 0.01, Fisher’s least significant difference method).

Next, we examined the amount of T required for stress relief using varying T concentrations with a CE/TA ratio of 2, as shown in Table 7. The results showed that a CE/TA ratio of 2 and a T concentration of 0.23 mM (40 mg/L) or higher significantly suppressed adrenal hypertrophy, even under the stressful conditions caused by confrontation rearing (Figure 7). Thus, the results suggest that a T concentration of 0.23 mM (40 mg/L) or greater is necessary for stress reduction.

The daily water intake of mice was approximately 8 mL, and the amount of T required for stress reduction could be calculated to be 10 mg/kg or more. However, as the water intake per body weight of mice is approximately eight times that of humans, it is necessary to consider species differences when extrapolating these results to humans.

Based on the results of the experiment using powdered green tea (Figure 1), the amount of T required for stress reduction was calculated to be 1.8 mg/kg and, so, the T requirement for a 50–60 kg human is estimated to be 100 mg. For a T concentration of 0.23 mM (40 mg/L) in solution, 2.5 L is estimated to be needed; meanwhile, for a 1.15 mM (200 mg/L) solution, 500 mL is needed.

## 3. Discussion

Two mechanisms are currently believed to be important for the stress-relieving effects of T: one is inhibition of the excessive release of the excitatory neurotransmitter glutamate via the glutamine transporter, while the other is the increased release of the inhibitory neurotransmitter GABA via Npas4 (Neuronal PAS domain protein 4). As T has a high affinity for the glutamine transporter; it has been proposed that T inhibits the uptake of glutamine and, consequently, the release of glutamate, an excitatory neurotransmitter [22]. Npas4 is a transcription factor that enhances many inhibitory GABA-releasing synapses to maintain the balance between excitation and inhibition in the nervous system [23]. We have found that T intake increases the expression of Npas4 in the hippocampus [5]. Thus, T maintains the balance between excitation and inhibition, thereby reducing stress.

As E and C cause a variety of effects, several mechanisms may be possible for their action in the brain. An important effect, however, may be that they act to counteract the action of T to enhance GABA release. It has been reported that E may modulate its activity through binding to the GABA_A_ receptor [24]. As GABA exerts its neuroinhibitory effects via the GABA_A_ receptor, E may antagonize the effects of T by acting on this receptor. Furthermore, C has been reported to have Ca^2+^-independent inhibitory effects on GABAergic inhibition [25] and also to increase glutamate levels by inhibiting adenosine A_1_ receptor [26]. On the other hand, *Nr4a1* (nuclear receptor 4a1, Nur77) expression was significantly increased in the hippocampus of mice in response to psychosocial stress, but its expression was significantly suppressed in A-fed mice, even under stress conditions [27]. Nr4a1 is a dopamine- and glutamate-dependent transcription factor known to be induced during stress [28]. This may suggest that A causes inhibition of excessive glutamate release. Alternatively, as endogenous NO has been reported to modulate GABA and glutamate release [29], A may enhance endogenous NO and reduce stress. These findings suggest that T and A reduce stress through maintaining a balance between excitatory glutamate and inhibitory GABA, whereas E and C inhibit the action of GABA.

Although green tea contains glutamic acid and small amounts of GABA, these cannot cross the blood–brain barrier and do not directly affect the balance between excitation and inhibition in the brain.

In this study, we examined tea leaves and their infusion conditions for stress-relieving effects. When mice ingested powdered green tea in feed, stress-relieving effects were observed for shaded green teas with CE/TA ratios of 1.33 to 2.74. On the other hand, green teas with CE/TA ratios of 5.80 and 14.53 (non-shaded green tea) showed no effect. This was in agreement with previous results [5], which showed that the stress-relieving effects of matcha teas occurred with CE/TA ratios of 2 or less.

The CE/TA ratio in the infused solutions was found to be lower than that in the tea leaves, suggesting that if the CE/TA ratio in the tea leaves is less than 2, the infused solution can be expected to have a stress-reducing effect when infused at a water temperature of 50–80 °C and infusion time of 2 to 3 min, according to the standard infusion method [30].

The results obtained when using a model green tea component solution showed that stress-relieving effects were obtained when the CE/TA ratio was 2 or less (Figure 6), and there was a minimum amount of T required (Figure 7); that is, the concentration of T must be greater than 0.23 mM (40 mg/L). T alone can be effective in reducing stress at concentrations of 30 µM or higher [20]; however, when C and E are present, at least eight times the concentration of T is required.

Based on the results of the experiment using powdered green tea (Figure 1), the amount of T required for stress reduction was calculated to be 1.8 mg/kg, and the estimated requirement for a human (50–60 kg body weight) was 100 mg. In previous studies in which C-reduced green tea was consumed by elderly and middle-aged people, a stress-reducing effect was observed at 60 mg/day of T [31]. Since the amount of C was reduced by about one-third in C-reduced green tea, the amount of T that would result in a CE/TA ratio of 2.0 was calculated to be exactly 60 mg. Considering these results, the estimated requirement of 100 mg of T in humans in the case of green tea obtained in this experiment is considered to be a reliable value. Table 4 indicates that, for green tea with a CE/TA ratio of 1.98 infused with water at 60 °C for 2 min, at least 250 mL would be required for stress reduction. Furthermore, when using an infused solution with 4 °C water for 15 h using tea leaves with a CE/TA ratio of 5.80, at least 725 mL would be required to reduce stress (Table 5). As these are feasible amounts for drinking, it can be expected that drinking infused green tea can reduce stress through the careful selection of tea leaves and brewing conditions.

The shaded green teas examined in this study were new leaves collected at the appropriate harvest time from tea plants grown under good growing conditions and with adequate shading. These teas had T levels of 18–32 mg/g. When new leaves were grown under favorable conditions and collected at the appropriate harvest time, T levels in the unshaded tea leaves were not low; however, T levels in the late harvested, slightly hardened tea leaves were very low. Furthermore, the T infusion efficiency decreased as the T content decreased. On the other hand, the efficiency of infusing C and E did not vary much among tea types.

Matcha is considered a typical green tea that is shaded from sunlight. However, about half of the matcha sold in Japan (76 samples) had a CE/TA ratio of 2 or less, while only one matcha sold overseas (67 samples) had a CE/TA ratio of 2 or less, and more than half had a ratio greater than 5 [5]. Although many shaded green teas have potential stress-reducing effects, it may be necessary in the future to share information such that consumers are aware of the CE/TA ratio in green teas.

A moderate level of stress is thought to be necessary for life and to have positive effects. However, when overloaded over a long period of time, stress is thought to cause the onset or worsening of various diseases, such as depression, mood disorders, cardiovascular diseases, and aging-related diseases [17,32,33,34]. During stress, the balance between excitation and suppression in the brain is disrupted, resulting in a state of heightened excitement. Therefore, substrates such as green tea that relieve initial excitement are important. We demonstrated the involvement of green tea components and infusion conditions in the associated stress relief effect.

## 4. Materials and Methods

### 4.1. Measurement of Tea Components

All green tea samples used in the study were produced in Mie Prefecture, Japan. Sample A was prepared by a green tea producer in Mie Prefecture. Samples B, C, and D were produced at the Mie Prefectural Agricultural Research Center for research purposes. Sample E was purchased commercially at a supermarket in Mie Prefecture.

The concentrations of amino acids, catechins, and caffeine in tea leaves and tea-infused solutions were determined through high-performance liquid chromatography. Free amino acid content was determined for eight amino acids, according to the method of Goto et al. [35], under the following measurement conditions. For the analysis of tea leaf samples, 50 mg of tea powder and 150 mg of polyvinylpyrrolidone were added to 50 mL of hot water with glycylglycine (final concentration 10 mg/L) as an internal standard, sonicated for 10 min, and extracted in a hot water bath at 80 °C for 30 min. The tea-infused solution was used as the sample, which was filtered. The guard column was Develosil ODS-HG-5 (φ4 mm × 10 mm, Nomura Chemical Co., Ltd., Aichi, Japan), and the separation column was Develosil ODS-HG-5 (φ4.6 mm × 150 mm, Nomura Chemical Co., Ltd., Aichi, Japan). The column temperature was 40 °C, and the injection volume of the induced sample was 10 μL. Mobile-phase A was 95% 5 mM citrate buffer (pH 6.0) and 5% acetonitrile, and B was 30% 5 mM citrate buffer (pH 6.0) and 70% acetonitrile at a flow rate of 1 mL/min. Elution was maintained at A:B = 95:5 from 0 to 5 min, with a gradient of A:B = 88:12 from 5 to 20 min and A:B = 62:38 from 20 to 30 min. Fluorescently labeled free amino acids were detected at an excitation wavelength of 340 nm and a fluorescence wavelength of 450 nm.

The four catechins and C content were measured using high-performance liquid chromatography under the following measurement conditions, according to the method of Yamaguchi et al. [36]. The mixture was mixed well, filtered, and diluted 10-fold with distilled water to form the sample for analysis. The guard column was Develosil ODS-HG-5 (φ4 mm × 10 mm, Nomura Chemical Co., Ltd., Aichi, Japan), and the separation column was Develosil ODS-HG-5 (φ4.6 mm × 150 mm, Nomura Chemical Co., Ltd., Aichi, Japan). The column temperature was 40 °C and the sample injection volume was 20 μL. The mobile phase was distilled water:acetonitrile:phosphoric acid (volume ratio 400:10:1) as liquid A, liquid B was liquid A:methanol (volume ratio 2:1), and the flow rate was set at 1 mL/min. Elution was maintained at A:B = 80:20 from 0 to 27 min, and A:B = 20:80 from 27 to 37 min. Detection was performed at 272 nm. Reagents used in chromatography were purchased from Kanto Chemical Co. Inc., (Tokyo, Japan). Standards used in chromatography were purchased from Fujifilm Wako Pure Chemicals Corporation (Osaka, Japan).

### 4.2. Preparation of Tea Sample for Mice

The intake of green tea was set at 100 mg/kg, based on the amount normally consumed by humans (6 g/60 kg). As mice (weighing approximately 30 g) eat approximately 5 g of food per day, the concentration of green tea in the powdered feed was set at 0.6 mg (green tea)/g (feed). The green tea leaves were ground into tea powder using a cyclone mill (CSM-F1, Shizuoka Seiki, Shizuoka, Japan) and mixed with powdered feed (CE-2; Clea Co. Ltd., Tokyo, Japan).

In the experiment in which green tea components were ingested as drinking water, the following four purified components were dissolved in water and fed ad libitum to mice: T (Suntheanine, Taiyo Kagaku Co., Ltd., Yokkaichi, Japan), E (Sunphenon EGCg, Taiyo Kagaku Co., Ltd. Yokkaichi, Japan), A, and C (Fujifilm Wako Pure Chemicals Corporation, Osaka, Japan).

### 4.3. Infusion Method

**Figure 8:**
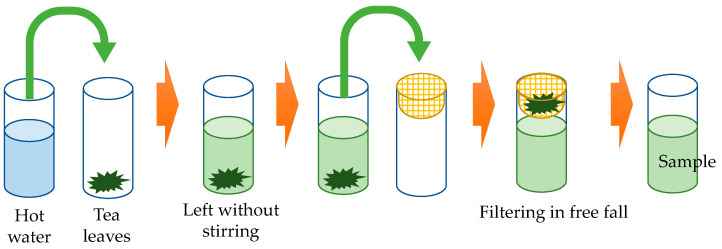
Tea leaf infusion method. Distilled water at various temperatures was placed together with weighed tea leaves in separate beakers heated to various temperatures. After the standing time, the tea leaves were removed using a net cup. The infused solution was then analyzed.

Tea leaves were leached as shown in Figure 8. Distilled water at various temperatures was prepared in a beaker, and this hot water was placed with the weighed tea leaves in another beaker heated to the respective temperature. After standing for each infusion time, the tea leaves were removed using a net cup, and the tea infused solution was used for the measurement. The infusion was carried out at room temperature and was not heated to maintain the temperature during the infusion process. The method used here was designed to imitate the common Japanese method of brewing tea using a Japanese teapot (Kyusu) and to eliminate variations in the brewing process.

### 4.4. Evaluation Methods for Stress Relief Effects

When stress is applied to the body, the HPA axis is activated in response to stress via excitatory signals, and the adrenal cortex releases glucocorticoid (corticosterone), resulting in adrenal hypertrophy [4]. We evaluated the stress-relieving effects of each green tea by comparing the degree of suppression of adrenal hypertrophy. Male ddY mice (Slc: ddY, 4 weeks old) were purchased from Japan SLC Co., Ltd. (Shizuoka, Japan) and reared under a controlled environment with a 12-h light/dark cycle (light period, 08:00–20:00), temperature (23 ± 1 °C), and relative humidity (55 ± 5%). Experiments were conducted with the approval of the University of Shizuoka Animal Experiment Committee (Approval No. 225354). All experimental protocols conformed to the U.S. National Institutes of Health guidelines for the care and use of laboratory animals.

**Figure 9:**
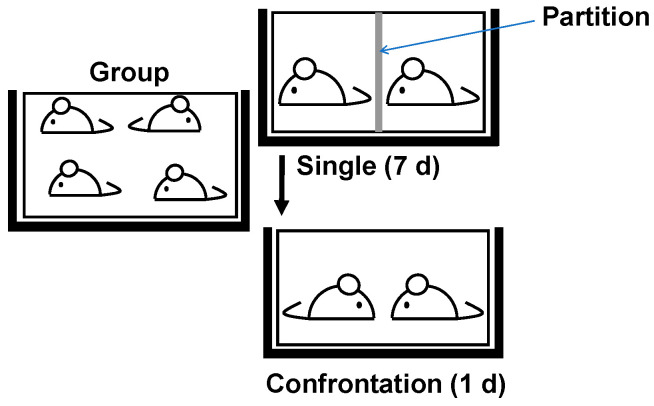
The control group was kept in groups of four animals per cage. The stress group was reared alone for 7 days, followed by 1 day of confrontation rearing with the partition board removed. These cages were placed in a Styrofoam box or cage cover in order to avoid social contact between cages.

Two mice were kept alone in partitioned cages (CLEA Japan Inc., Tokyo, Japan) for one week to establish territoriality, as shown in Figure 9. When the partitions were removed on day 7, the two mice were stressed by the presence of other mice in own territory. Since adrenal hypertrophy is maximal after 24 h [20], they were dissected one day later; adrenal glands were removed, and the total wet weight (left and right) was precisely measured. A dominant–subordinate relationship was observed in confrontation-reared mice, but neither the submissive nor dominant mice suffered any serious injuries. Body weights did not differ between the confrontation rearing. Significant adrenal hypertrophy persists for more than a week in ddY mice used in this study. It persisted even longer in the strain that is vulnerable to stress. Confrontation stress has been observed in all strains of male mice we have examined. The control group was fed a powdered or solid diet (CE-2, CLEA Japan Inc., Tokyo, Japan) without green tea. Group-fed (4 mice/cage) mice, which is considered less stressful for mice, were used for comparison (i.e., as a control) (Figure 9).

These cages were placed in Styrofoam boxes or cage covers (Natsume Seisakusho Co., Ltd., Tokyo, Japan) in order to avoid social contact between cages. The groups, including the control, were tested twice with confrontation and group-reared mice (4 mice per group), and data from a total of 8 mice were compiled to evaluate the stress-relieving effects of each green tea.

### 4.5. Statistical Analysis

Wet weights of adrenal glands in each group are presented as mean ± standard error. Statistical analysis was performed using one-way analysis of variance, and statistical significance was set at *p* < 0.05. Confidence intervals and significance of differences in means were estimated according to Fisher’s least significant difference method.

## 5. Conclusions

The amino acids T and A in tea leaves exhibit excellent stress-relieving properties. On the other hand, C and E, the major components in green tea, antagonize their effects. When tea leaves with a CE/TA ratio of 5.80 (one of the Sencha green teas grown under sunlight) were consumed as a powder, no stress-relieving effect was observed. However, when the tea leaves were infused with hot water at 60 °C or cold water at 4 °C, the CE/TA ratio of the infused solution was less than 2, indicating that the infused solution could have stress-relieving effects. It was also found that the T concentration in the infused solution should be at least 0.23 mM. These results suggest that the CE/TA ratio in tea leaves and their infusion conditions should be carefully selected if green tea is expected to have stress-relieving effects.

## Figures and Tables

**Table 1:** Tea components in tea leaves.

Tea Component	Sample A		Sample B		Sample C		Sample D		Sample E	
	Shaded “Seimei”		Shaded “Yabukita”		Shaded “Yabukita”		Non-Shaded Soft New Leaves		Non-Shaded Hard New Leaves	
	mg/g	μmol	mg/g	μmol	mg/g	μmol	mg/g	μmol	mg/g	μmol
Theanine (T)	32.29	185.4	27.51	157.9	18.07	103.7	7.46	42.8	3.26	18.7
Arginine (A)	6.77	38.9	4.42	25.4	2.19	12.6	0.76	4.4	0.30	1.7
Glutamine	12.68	86.8	7.82	53.5	4.93	33.7	1.51	10.3	0.55	3.8
Serine	1.55	8.4	1.44	7.8	0.86	4.6	0.37	2.0	0.24	1.3
Asparagine	1.30	9.8	1.20	9.1	0.39	3.0	0.02	0.2	0.06	0.5
Glutamic acid	6.04	41.1	3.98	27.1	3.27	22.2	1.47	10.0	1.27	8.6
Aspartic acid	6.50	48.8	4.72	35.5	2.74	20.6	1.04	7.8	1.10	8.3
Caffeine (C)	33.66	173.3	37.36	192.4	29.86	153.8	21.32	109.8	22.78	117.3
EGCG (E)	57.47	125.4	77.94	170.0	75.51	164.7	75.12	163.9	82.36	179.7
EGC	28.93	94.5	18.88	61.6	31.73	103.6	47.24	154.2	52.15	170.3
ECG	12.36	27.9	12.91	29.2	11.94	27.0	12.54	28.3	14.27	32.3
EC	8.83	30.4	6.65	22.9	7.99	27.5	10.46	36.0	10.85	37.4
CE/TA molar ratio		1.33		1.98		2.74		5.80		14.58

ECG; epicatechin gallate.

**Table 2:** Tea components in each tea-infused solution.

Tea Component	CE/TA Molar Ratio in Tea Leaves							
	1.33 (Sample A)		1.98 (Sample B)		5.80 (Sample D)		14.58 (Sample E)	
	mg/L	mM	mg/L	mM	mg/L	mM	mg/L	mM
Theanine (T)	455.8	2.62	398.6	2.29	97.4	0.56	18.6	0.11
Arginine (A)	70.9	0.41	53.5	0.31	4.6	0.03	0.7	0.004
Glutamine	181.6	1.24	107.9	0.74	18.1	0.12	2.9	0.02
Serine	22.4	0.21	21.0	0.20	9.1	0.09	1.8	0.02
Asparagine	18.2	0.14	18.6	0.14	0.4	0.003	0.5	0.004
Glutamic acid	94.8	0.64	61.4	0.42	22.8	0.15	8.6	0.06
Aspartic acid	104.0	0.78	70.5	0.53	14.2	0.11	6.6	0.05
Caffeine (C)	389.3	2.00	431.3	2.22	172.8	0.89	137.0	0.71
EGCG (E)	213.5	0.47	299.1	0.65	224.9	0.49	233.7	0.51
EGC	276.8	0.90	207.6	0.68	387.8	1.27	293.5	0.96
ECG	39.8	0.002	34.1	0.002	36.3	0.002	37.4	0.002
EC	88.3	0.30	55.9	0.19	84.6	0.29	65.9	0.02
CE/TA molar ratio in infused solution	0.82		1.11		2.36		10.99	

Five grams of tea leaves were infused with 200 mL water at 60 °C for 2 min.

**Table 3:** Elution efficiency of each tea component.

	Elution Efficiency			
CE/TA in Tea Leaves	1.33 (Sample A)	1.98 (Sample B)	5.8 (Sample D)	14.58 (Sample E)
Theanine	0.565	0.580	0.522	0.228
Arginine	0.419	0.484	0.242	0.093
Glutamine	0.573	0.552	0.479	0.211
Serine	0.578	0.583	0.984	0.300
Asparagine	0.560	0.620	0.800	0.333
Glutamic acid	0.628	0.617	0.620	0.271
Aspartic acid	0.640	0.597	0.546	0.240
Caffeine	0.463	0.462	0.324	0.241
EGCG	0.149	0.154	0.120	0.114
EGC	0.383	0.440	0.328	0.225
ECG	0.129	0.106	0.116	0.105
EC	0.400	0.336	0.324	0.243

These elution rates were based on the values in Table 1 and Table 2 and were obtained when 5 g of tea leaves were infused with 200 mL of hot water at 60 °C for 2 min.

**Table 4:** Effect of tea leaf volume on the CE/TA ratio.

Elution Condition	100 mL of Water at 60 °C	
Tea Leaves	2.5 g	5 g
Theanine (T)	398.6 (mg/L)	783.7 (mg/L)
Arginine (A)	53.5	111.6
Glutamine	107.9	209.8
Serine	21.0	39.2
Asparagine	18.6	34.5
Glutamic acid	61.4	115.5
Aspartic acid	70.5	150.3
Caffeine (C)	431.3	830.7
EGCG (E)	299.1	602.2
EGC	207.6	414.2
ECG	34.1	56.0
EC	55.9	113.1
CE/TA molar ratio	1.11	1.09

Here, 2.5 g or 5 g of tea leaves with a CE/TA ratio of 1.98 (sample B) were infused with 100 mL of water at 60 °C for 2 min.

**Table 5:** Effect of water temperature on the elution of each component.

Component (mg/L)	Infusion Condition	
	80 °C, 1 min	4 °C, 15 h
Theanine (T)	93.6	138.0
Arginine (A)	4.7	8.8
Glutamine	17.1	25.7
Serine	8.7	11.0
Asparagine	0.2	0.5
Glutamic acid	21.1	30.7
Aspartic acid	13.3	18.1
Caffeine (C)	237.6	210.5
EGCG (E)	291.2	169.2
EGC	406.4	673.8
ECG	47.5	28.3
EC	92.2	124.1
CE/TA molar ratio	3.29	1.72

Five grams of tea leaves with CE/TA ratios of 5.80 (sample D) were infused in 200 mL of water at 4 °C for 15 h, or 2.5 g of tea leaves in 200 mL of water at 80 °C for 1 min.

**Table 6:** Concentrations and CE/TA ratios of the four selected components.

Components (mM)	CE/TA Molar Ratio				
	0.5	1.0	2.0	4.0	10.0
Theanine (T)	4.6	2.3	1.15	0.6	0.23
Arginine (A)	0.6	0.3	0.15	0.05	0.03
EGCG (E)	0.6	0.6	0.6	0.6	0.6
Caffeine (C)	2.0	2.0	2.0	2.0	2.0

**Table 7:** Compositions ensuring a CE/TA ratio of 2.0 under different theanine concentrations.

Component (mM)	CE/TA Molar Ratio 2.0				
Theanine (T)	1.15	0.6	0.23	0.115	0.06
Arginine (A)	0.15	0.05	0.03	0.015	0.005
EGCG (E)	0.6	0.3	0.12	0.06	0.03
Caffeine (C)	2.0	1.0	0.4	0.2	0.1

## Data Availability

The data presented in this study are available upon request from the corresponding author.
